# Caring for the allergic patient – WAO White Book on Allergy 2026 - 1^☆^

**DOI:** 10.1016/j.waojou.2026.101369

**Published:** 2026-04-03

**Authors:** Roy Gerth van Wijk

**Affiliations:** Section of Allergology and Clinical Immunology, Department of Internal Medicine, Erasmus Medical Center, Rotterdam, Netherlands

**Keywords:** Allergy care, Education, Training requirements, Multidisciplinary, Shared decision-making

## Abstract

Optimal care for allergic patients requires several key conditions. First, patients should have access to well-educated and well-trained specialists and primary care physicians. Training and education should be competence-based, for example, following the principles of the CanMEDS framework tailored to the field of allergy. Physicians should not only be medical experts but also communicators and collaborators, leaders and health advocates, scholars, and professionals. Well-designed training programs should aim at standards of care and harmonisation of clinical practice. Also, the ethical aspects of care should be taken into account.

Second, recognizing that allergy is a systemic disease that transcends the boundaries of medical disciplines, collaboration among healthcare professionals is essential for high-quality allergy care. This necessitates facilities capable of delivering multidisciplinary care, ideally through the establishment of well-organized allergy centres. Not only physicians but also nurses, dieticians, psychologists and pharmacists should play a role.

Given that interventions and treatments can have a significant impact, effective communication and collaboration with the patient are essential. To give the patient a voice, one can use shared decision-making. Shared decision making may enhance adherence, outcomes, and patient satisfaction.

**Key statements**

• Allergic diseases are complex, often involving multiple body systems, and affect patients in complex ways.
• Caring for the allergic patient therefore requires comprehensive education and training of physicians involved in allergy.
  ○ This should include a focus on competencies.
  ○ This is true whether or not the country has specialists in allergology.
• Optimal care requires multidisciplinary coordination.
• Shared decision-making allows the patient to take an active role in care and treatment and may increase adherence.

## Introduction

Allergic diseases are characterized by high prevalence, chronicity, and high disease burden. Allergic patients are not only affected by their symptoms, but they must also take relevant environmental measures such as removing pets or implementing other changes indoors, or implementing diets and coping with the effects of food allergies on their social lives. Drug allergies may hamper the optimal treatment of other medical conditions. Long-term pharmacotherapy and/or allergen immunotherapy may be required. Because allergy is a systemic disease with manifestations in different organs, doctors need a comprehensive, holistic approach to their treatment of allergic diseases. For successful management of allergic disorders, the full cooperation of the allergic patient is needed.

The question is how to achieve optimal care for allergic patients. The backbone of high-quality care is the availability of well-educated and trained specialists and primary care physicians. Care also has to be organized in a way that allergic patients have access to well-established allergy services. Good collaboration between healthcare workers is a key factor for achieving the best possible care. Finally, caring for the allergic patient means that the patient has a voice in his or her disease's management. Healthcare has to be tailored to the needs of the patient.

## Education and training

Traditionally, the core elements of education and training in the specialty of allergology and clinical immunology are theoretical knowledge and practical skills. The content of these programs is based on internationally accepted guidelines and evidence-based medicine. For instance, the recently established European Training Requirements (ETRs) approved by the European Union of Medical Specialists (UEMS) in 2019^1^ covers knowledge and skills in all relevant areas such as allergic rhinoconjuctivitis; asthma; atopic dermatitis; urticaria; angioedema; anaphylaxis; food, drug, and venom allergy; and other conditions, diagnostic procedures, and specific interventions such as allergen avoidance, allergen immunotherapy, and prevention of allergy. The ETRs also include education and training in immune deficiencies and autoimmune diseases for those countries that recognize the specialty of both allergology and clinical immunology. However, the scope of the UEMS ETRs is much broader and focused on competencies. UEMS values professional competencies as “the habitual and judicious use of communication, knowledge, technical skills, clinical reasoning, emotions, values, and reflection in daily practice for the benefit of the individual and community being served.”^2^

Key competencies for optimal care are those described in the CanMEDS framework^3^ (ie, medical expert, communicator, collaborator, leader, health advocate, scholar, and professional (See Table 1).^4^ These competencies should apply to all physicians in all disciplines and, hence, to allergy specialists and subspecialists. Taking these competencies as guidance, the ETRs for allergology define the roles for allergists (Table 1).^4^

**Table 1:** CanMEDs competencies and the resulting roles of the allergist^4^

A medical expert

*CanMEDs competencies* • Practice medicine within their defined scope of practice and expertise. • Perform a patient-centered clinical assessment and establish a management plan. • Plan and perform procedures and therapies for the purpose of assessment and/or management. • Establish plans for ongoing care and, when appropriate, timely consultation. • Actively contribute, as an individual and as member of a team providing care, to the continuous improvement of health care quality and patient safety.
*Roles of the allergist* • The allergist has the necessary knowledge and skills to adequately manage patients with allergic, hypersensitivity, and certain non-allergic diseases. • Because allergology is multidimensional and multidisciplinary, the specialist has relevant knowledge and skills beyond single-organ systems and specific age groups. • The allergist is skilled in clinical reasoning. • Diagnostic and therapeutic procedures and interventions are carried out according to the principles of evidence-based and cost-effective medicine. • Unnecessary or harmful investigations or treatment need to be avoided. • The specialist follows national and international guidelines. • The specialist is aware of his or her strengths but also recognizes the limitations of knowledge and skills and refers the patient to other specialists when appropriate.
A communicator
*CanMEDs competencies* • Establish professional relationships with patients and their families. • Elicit and synthesize accurate and relevant information, incorporating the perspectives of patients and their families. • Share health care information and plans with patients and their families. • Engage patients and their families in developing plans that reflect the patient's health care needs and goals. • Document and share written and electronic information about the medical encounter to optimize clinical decision-making, patient safety, confidentiality, and privacy.
*Roles of the allergist* • The allergist can listen carefully and get and synthesize relevant information from patients and families. • He or she is able to establish an effective dialogue with patients and gives appropriate feedback. • The specialist understands that factors such as age, gender, disability, ethnicity, and cultural and social background may have an influence on the patient's history, relationships, and ability to comply with a therapeutic intervention. • The specialist can guide and educate patients regarding disease, its treatment, risk factors, and prevention of disease. • The allergist establishes a relationship with trust, understanding, and compassion. Shared decision-making is a part of the therapeutic relation with the patient. • The allergist is able to communicate effectively with colleagues and other health care providers.
A collaborator
*CanMEDs competencies* • Work with physicians and other colleagues in the health care professions to promote understanding, manage differences, and resolve conflicts. • Hand over the care of a patient to another health care profession to facilitate continuity of safe patient care.
*Roles of the allergist* • Taking the systemic nature of allergy and allergic diseases into account, the allergist will work at the interface of different medical disciplines. • He or she is able to act in multidisciplinary settings. • The specialist is able to understand and value the roles, opinions, and contributions of other health care professionals. If appropriate, care of patients can be safely handed over to colleagues and other health care professionals.
A leader
• *CanMEDs competencies* • Contribute to the improvement of health care delivery in teams, organizations, and systems. • Engage in the stewardship of health care resources. • Demonstrate leadership in professional practice. • Manage career planning, finances, and human resources in a practice.
*Roles of the allergist* • The allergist has a vision about his or her role and position in the health care organization. • The allergist is responsible for the care of allergic patients and aims to take the lead in matters regarding allergy. • The allergist aims to optimize allergy care within the framework of health care. • At a management level the allergist is able to lead his own practice and supporting personnel. • The allergist has management skills in organization and coordination of care.
A health advocate
*CanMEDs competencies* • Respond to an individual patient's health needs by advocating with the patient within and beyond the clinical environment. • Respond to the needs of the communities or populations they serve by advocating with them for system-level change in a socially accountable manner.
*Roles of the allergist* • The allergist will advocate health promotion for allergic patients both in their own practice and at a general level. • The allergist will be able to identify determinants of health for a management and prevention plan and ensure that patients are able to access appropriate health and social services in the management of individual patients. • The specialist will identify patient groups at risk of allergic disease and its complications and apply available knowledge about primary and secondary prevention. • The allergist will identify issues and opportunities for improving allergy health care at a general level.
A scholar
*CanMEDs competencies* • Engage in the continuous enhancement of their professional activities through ongoing learning. • Teach students, residents, the public, and other health care professionals. • Integrate best available evidence into practice. • Contribute to the creation and dissemination of knowledge and practices applicable to health.
*Roles of the allergist* • The allergist must undertake a lifelong learning in allergology. • The specialist must contribute to the education of students, patients, colleagues, and the general public. • He or she should appraise the sources of medical information critically, understand the importance of ongoing research, and participate in and contribute to research.
A professional
*CanMEDs competencies* • Demonstrate a commitment to patients by applying best practices and adhering to high ethical standards. • Demonstrate a commitment to society by recognizing and responding to societal expectations in health care. • Demonstrate a commitment to the profession by adhering to standards and participating in physician-led regulation. • Demonstrate a commitment to physician health and well-being to foster optimal patient care.
*Roles of the allergist* • The allergist is committed to delivering the highest care possible to the allergic patient. • Care should be carried out according to medical ethical standards, including the use of informed consent, advanced directives, research ethics, and respect for the autonomy of the patient.

From this table, it can be seen that the demands for a specialist who wishes to deliver optimal allergy care and who is caring for the allergic patients are high. The requirements go beyond the classical approach of mastering theoretical knowledge and clinical skills. The UEMS has incorporated the development of Entrustable Professional Activities (EPAs) as the highest level of clinical skills. EPAs are professional activities that form the day-to-day work of the healthcare professional. A supervisor only entrusts a student with a professional activity to perform independently if the student has shown that they are sufficiently competent to do so.^5^ For example, the ETR Allergology describes an EPA of “Diagnosis and treatment of allergic disorders”, which includes all of the above-mentioned CanMED roles.^4^

A recent World Allergy Organization (WAO) publication on allergy education and training for physicians proposes levels of competencies divided into core, additional, and specialist competencies to be mapped onto different infrastructures irrespective of the presence or absence of an allergology (sub)specialty.^6^ Core competencies comprise the amount of knowledge and clinical skills necessary for recognition and interpretation of allergic diseases. Additional competencies are required to manage patients with allergies more independently and to provide advice. Specialist competencies focus on complex diseases and management strategies. In addition, physicians should be aware of the multidisciplinary facets of allergy care, including patient education, psychological care, and dietetic interventions. Special attention has to be paid to the ethical standards of allergy care. The allergic patient should be protected against unethical practices of over-investigation and unorthodox, non-validated diagnostic procedures and therapeutic interventions.

## Organization of care

The allergic patient should have access to healthcare providers experienced in allergic diseases. In many countries, however, allergology is not recognized as a specialty or a subspecialty. The proposal to establish levels of competencies attempts to overcome the heterogeneity in allergy services worldwide, as mapping of competencies to different training and healthcare systems is not dependent on the presence of a specialty in allergology.^6^ A European Academy of Allergy and Clinical Immunology (EAACI) position paper aimed to determine the roles and responsibilities of physicians in allergy care.^7^ Whereas the organ-based specialist can use standard allergy diagnostic tests, laboratory and function tests relevant to the field, and organ-based therapy, the allergist/clinical immunologist can manage multi-organ allergic disease; can implement allergen immunotherapy; and is able to coordinate care at the interface between the patient's condition, the preventive strategies, and the available treatment options. The allergist should be able to use new techniques. Partnership between organ-based physicians and allergists is highly important. Primary care physicians should be able to treat patients with clear allergies and mild symptoms. They can be seen as a gate-keeper because they should refer more complex cases to the allergist and/or organ-based specialist. To bridge the gap between the need and availability of experienced and accredited physicians who can provide optimal care for allergic patients, the EAACI Specialty Committee proposed the minimum requirements for training and certification of subspecialists in allergology.^8^ This document describes the required theoretical knowledge, skills, competencies, and training facilities (personnel and institution). The subspecialist, as described in this article, should ideally have the necessary competencies to provide good-quality care to patients in an environment that lacks the full allergology specialists or tertiary pediatric subspecialists in allergy.

Collaboration between healthcare professionals is a prominent part of allergy care. Daniels et al elaborate this in a comprehensive model, in which not only doctors but also nurses, dieticians, psychologists, and pharmacists play a role.^9^ Nurses can provide care and education to patients from a holistic perspective. They can also contribute to administration and research. Dietitians play a key role in the treatment of food allergies. Psychologists provide help to implement coping strategies to support the patient and family. Pharmacists can advise on medicines, check correct use, and supervise correct administration techniques. In addition, patient groups also have a place in this model. By offering peer mentors and evidence-based resources, they can support patients in managing their condition.

Whereas a multidisciplinary approach is key in the care of the allergic patient, optimal care can be hampered by a lack of coordination and confusion about the different roles and responsibilities of the players in the field. A way to overcome these problems is the development and implementation of integrated care pathways (ICPs).^9^ Unfortunately, although effective in the context of hospital care, acute conditions, and routine surgery, several studies identified flaws perceived by allergic patients involved in ICPs.^10^^,^^11^ Potential points for improvement were access to care, communication, information, support,^10^ and a better structure and extension of collaboration with such other professionals as pharmacists, nurses, and occupational physicians.^11^ It is therefore recommended to implement ICPs according to an evidence-based methodology with equal positions for all stakeholders, particularly for patients and their caregivers.^9^

## The voice of the patient: shared decision-making

In the last few decades, the role of the patient as an active partner in disease management has been emphasized. The phrase “patient-centered care” was coined in 1988 to shift the focus away from diseases and back to the patient and family. Patient-centered care has been defined as “care that is respectful of and responsive to individual patient values” and that ensures “that patient values guide all clinical decisions.”^12^ Shared decision-making is a way to engage patients. Already in 1990, Eddy stated that “an intervention should be considered as a standard only if there is virtual unanimity among patients about the overall desirability … of the outcomes.”^13^ Shared decision-making is particularly important in complex, chronic disorders like food allergy.^14^ For instance, the complexity of the diagnosis, the need for dietary intervention, early introduction of food, and the possibilities and limitations of oral immunotherapy require understanding and commitment of the patients and caregivers. Shared decision-making entails effective communication, including closely listening to and respecting patient concerns and wishes, as a prerequisite for establishing adherence to allergen immunotherapy.^15^ Shared decision-making for asthma has been shown to improve adherence, outcomes, and patient satisfaction with care.^16^ Patient decision aids are useful tools for shared decision-making and have recently been developed for allergen immunotherapy, severe asthma, and atopic dermatitis.^16^, ^17^, ^18^

## Conclusion

Caring for the allergic patient requires comprehensive education and training of physicians involved in allergy. This training goes beyond the classical knowledge and skills. Competencies-based training aims to promote collaboration between healthcare workers and to keep the focus on patient-centered care. Working in multidisciplinary teams that include all relevant stakeholders may lead to better care. Integrated care pathways may be helpful in health care processes, but further research is needed to meet the needs of the patient. To engage allergic patients, shared decision-making should be part of the clinical practice.

## Abbreviations

CanMEDS, Canadian Medical Education Directives for Specialists; EAACI, European Academy of Allergy and Clinical Immunology; EPA, Entrustable Professional Activities; ETR, European Training Requirements; ICP, Integrated Care Pathways; UEMS, Union Européenne des Médecins Spécialistes; WAO, World Allergy Organization

## Ethics statement

As a review article, this work did not require ethics approval.

## Availability of materials

Not applicable.

## Author contributions

R Gerth van Wijk is the sole author of the work.

## Author approval to publish

I have reviewed the final version and agree to publication in WAO Journal.

## Funding

No funding to report.

## Conflict of interest statement

No conflicts to report.
